# On the Diseases, Injuries, and Malformations of the Rectum and Anus

**Published:** 1859-01

**Authors:** 


### NORTH AMERICAN

### MEDICO-CHIRURGICAL REVIEW.

JANUARY, 1859.

### Analytical and Critical Reviews.

ART. I.—*On the Diseases, Injuries, and Malformations of the Rectum and Anus, with remarks on habitual Constipation.* By T. J. ASHTON, Surgeon to the Blenheim Dispensary, Fellow of the Royal Medico-Chirurgical Society, Member of the Pathological Society of London, Corresponding Fellow of the Pathological Society of Montreal, Member of the Council of the Harveian Society, formerly House-Surgeon to University College Hospital. Second edition, octavo, pp. 396. John Churchill: London, 1857.

THE first edition of this work appeared in 1854. This second edition differs but little from its predecessor; some additional cases have been introduced in further illustration of several of the subjects, particularly of imperforate anus and rectum, which subject had not been sufficiently noticed in the other; in some of the chapters a few paragraphs have been inserted, and in one of them, the ninth, a few have been omitted. Three figures, moreover, have been introduced, for the purpose of representing the speculum which may be used in rectal examinations. The author says, and we believe with justice, "That he has found no reason to alter the arrangement of the contents, nor, as a result of more extended experience, to deviate from the principles of treating the various diseases propounded in the first edition."

The notices of this publication in numerous medical journals, and, in fact, the rapid exhaustion of its first edition, show the high estimation in which it is held by the medical profession. It well deserves its reputation, for it is undoubtedly the most complete work in the English language upon the subject of which it treats.

But the reputation of this work does not belong entirely to Mr. Ashton, and in appropriating it altogether to himself, he takes what he cannot rightfully claim. Much of the "good name" he bears he has taken from another, and, what adds to the greatness of the offence, great as it already is, from a deceased medical brother. Such a charge as this is most serious; we make it with reluctance, but, at the same time, without hesitation.

In several medical journals, but particularly in the *American Journal of Medical Sciences* for April, 1858, it has been noticed that Mr. Ashton is under great obligations to the treatise of the late Dr. Bushe, of New York; but the extent of these obligations has never been sufficiently known, or never properly pointed out. One journal seems to think that it is quite proper, indeed quite commendable, in Mr. Ashton, to appropriate to his own use the labors of another. The *British and Foreign Medico-Chirurgical* for July, 1857, says, "We have heard it objected that it (his work) has been too much based upon the work of the late Dr. Bushe; but even if this were true, which we do not think it is, it would in our eyes constitute rather a merit than a reproach." We must look upon this as a very strange, not to say a very loose, way of regarding such a matter.

All that Mr. Ashton says in regard to his indebtedness to Dr. Bushe's work is this:—

"When the treatment of these affections first occupied my attention, I greatly felt the want of a book to which I could refer for information on some of the more obscure and difficult cases. In the systematic works on surgery I found the subject but superficially treated; and though several excellent monographs have been published, the authors have confined their observations to certain of the affections only; among these stand pre-eminently the writings of Mr. Pott, Mr. Copeland, and Mr. Syme. The treatise of Dr. Bushe, of New York, who unfortunately died soon after its publication, is the only one with which I am acquainted that embraces the whole subject, and from it I have gained much valuable information."

We quote the whole passage that the reader may judge for himself, with correctness, of the exact amount of indebtedness acknowledged.

Dr. Bushe's work, which is entitled "A Treatise on the Malformations, Injuries, and Diseases of the Rectum and Anus," the same as that of Dr. Ashton, the order of the subjects being transposed, appeared in 1836. In his advertisement the author states that from pressure of business and declining health he had been more than a year in correcting the proof; and he adds that he hopes to bring out another edition, in which the mistakes and omissions of the first should be corrected. He died, however, soon after its publication; *unfortunately*, Mr. Ashton says; if so, it is more true of Dr. Bushe than of himself.

It is very difficult, indeed in a limited space it is at times impossible, to show how far one author is indebted to another. In some instances, as in that of M. Pró, of Lima, about whose plagiarism from Mr. Thompson the English journals are making so much ado, where for several pages the original is copied word for word, nothing is more easy;* but when the copier has acted with more cunning, and in a whole work, has changed the order of chapters, altered the arrangement of paragraphs, incorporated long foot-notes in the text, etc. etc. the task of showing the extent of the plagiarism is very difficult, however satisfied the reviewer may be himself of the truthfulness of his opinion. We shall endeavor, however, in this case, as we are indeed bound to do, after what we have said, to point out instances of plagiarizing on the part of Mr. Ashton, in sufficient number and of sufficient importance to justify all that we have said.

To make an accusation of this kind is very disagreeable to us, and the task of proving its correctness is by no means a pleasant one. It was impossible, however, for us to notice Mr. Ashton's book without making it, and having made it, we must justify it. Although in the eyes of some, it may, alas! be a merit rather than a reproach to borrow without acknowledgment from the works of others, in the eyes of many such conduct is considered most reprehensible. M. Pró, as we have said in our note, was dealt with very severely, and between M. Pró and Mr. Ashton we see a great difference in respect to skill, but no other—unless it be that of country.

Let us commence our task by comparing the list of contents in the two volumes.

| In Dr. Bushe's it is as follows:— | In Mr. Ashton's it is as follows:— |
|---|---|
| 1. Anatomy of the rectum. | 1. Irritation and itching of the anus. |
| 2. Functions of the rectum. | 2. Inflammation and excoriation of the anus. |

* In this much talked of plagiarism twenty-three pages of M. Pró's Memoir were copied, translated word for word, as stated, from Mr. Thompson's work. The English journals expressed great indignation, and the French Society of Surgery, of which M. Pró was a corresponding member, expelled him, and sent a representation of the whole affair to the Medical School of Lima, where he was professor, and demanded the notice of his expulsion in all medical journals. It is somewhat worthy of remark that the English journals warn continental writers not to dare to steal from English productions any longer, literary intercourse, they say, is too great, they must certainly be detected, when the plagiarism was first pointed out in this country, in the *American Journal of Med. Sciences* for April, 1858, as Mr. Thompson warmly acknowledges in a private letter to the writer of the review in which the matter is noticed.

| | |
|---|---|
| 3. Malformations of the rectum and anus. | 3. Excrescences of the anal region. |
| 4. Foreign bodies in the rectum. | 4. Contraction of the anus. |
| 5. Inflammation of the rectum. | 5. Fissure of the anus and lower part of the rectum. |
| 6. Inflammation and excoriation of the anus. | 6. Neuralgia of the anus and extremity of the rectum. |
| 7. Inflammation of the rectum and anus arising from the application of gonorrhœal matters. | 7. Inflammation of the rectum. |
| 8. Fissure of the anus. | 8. Ulceration of the rectum. |
| 9. Neuralgia of the extremity of the rectum. | 9. Hemorrhoidal affections. |
| 10. Spasmodic contraction of the sphincter ani. | 10. Enlargement of the hemorrhoidal veins. |
| 11. Ulceration of the rectum. | 11. Prolapsus of the rectum. |
| 12. Venereal ulceration of the rectum. | 12. Abscess near the rectum. |
| 13. Affections called hemorrhoidal. | 13. Fistula in ano. |
| 14. Enlargement of the hemorrhoidal veins. | 14. Polypi of the rectum. |
| 15. Prolapsus of the rectum. | 15. Stricture of the rectum. |
| 16. Relaxation of the anus. | 16. Malignant diseases of the rectum. |
| 17. Relaxation of the anus with invagination of the mucous membrane. | 17. Injuries of the rectum. |
| 18. Itching of the anus. | 18. Foreign bodies in the rectum. |
| 19. Excrescences about the anus. | 19. Malformations of the rectum. |
| 20. Polypi of the rectum. | 20. Habitual constipation. |
| 21. Abscess near the anus. | |
| 22. Fistula in ano. | |
| 23. Contraction of the anus. | |
| 24. Stricture of the rectum. | |
| 25. Carcinomatous degeneration of the rectum. | |

From these lists it will be seen that Mr. Ashton's book does not contain any separate chapters upon the anatomy and functions of the rectum, upon inflammation and excoriation of the anus, upon inflammation of the rectum and anus arising from the application of gonorrhœal virus, upon relaxation of the anus, and upon relaxation of the anus with invagination of the mucous membrane, while it contains one that Dr. Bushe's does not, upon habitual constipation. The other chapters are upon the same subjects; their order, however, being altered.

Let us now take the first chapter of Mr. Ashton's work, and see how much it resembles the nineteenth chapter of Dr. Bushe's, which is upon the same subject.

Dr. Bushe says :—

"The itching is often very distressing on going to bed, and not unfrequently prevents sleep for several hours;—the skin about the anus becomes thick, dense and furrowed;—the furrows assume a radiated direction, and converge in the anus; they vary in number from six to ten, and are from one-quarter to an inch in length. Though pretty deep, they are generally free from ulceration in those who are cleanly in their persons; but when ablution is not daily resorted to, they become impacted with irritating secretions, which create inflammation, excoriation, and even ulceration of the lining integument.

"The late Dr. Lettsom thought that the pruriginous state of the anus prevented the occurrence of more serious diseases.

"In the treatment of this obstinate and very troublesome disease * * provided the habits are sedentary, and the health delicate, exercise in the open air, alterative and chalybeate medicines, with a well-regulated nutritious diet, will be necessary. * * Should the patient have been in the habit of partaking of highly seasoned food, and drinking liquors freely, he may be put upon a vegetable diet, and restricted to water. * *

Much comfort and advantage will be derived from two or three warm baths each week. The local applications which I have found the most useful are the yellow wash, lead-water, and laudanum. * *

I have, however, observed far more

Mr. Ashton says :—

"The itching is often most distressing on getting warm in bed, and frequently prevents the patient sleeping until he is completely worn out. The skin around the anus will become thickened and furrowed; the furrows assuming a radiated direction diverging from the centre of the anus. They vary in number and length, and though often deep, are generally free from ulceration, if due attention to cleanliness is observed; but should this have been neglected, and irritating secretions have accumulated, inflammation will be induced, followed by excoriation and ulceration.

"Some authors think that a pruriginous state of the anus ought not to be interfered with, as it wards off more serious diseases.

"In the treatment of this very obstinate and troublesome disease * * should the patient be delicate and his habits sedentary, plain and nutritious food will be necessary, conjoined with proper exercise and the administration of alterative, tonic, and chalybeate medicines; but if the contrary be the case, and he has been accustomed to indulge himself in highly-seasoned dishes, and to partake freely of wine and spirituous liquors, he must be restricted to a vegetable diet, and the quantity of stimuli considerably reduced, if not altogether disallowed. * * Much advantage as well as comfort will be derived from the use of the warm bath every second or third day. The local remedies that will be found most useful are lotions containing nitrate of lead with wine of opium, lime-water, and calomel, or the bichloride of mercury. * *

But that which will frequently be benefit to arise from rubbing the surface lightly over with the nitrate of silver so as to produce a slight exfoliation of the skin." * *

found most serviceable, is brushing the part over with a solution of nitrate of silver so as to produce a slight exfoliation of the skin."

In this short chapter of five and a half pages, containing not more than 140 words to the page, a great part has been taken, with very little alteration, as we have shown; some more has been taken and a good deal altered; a very little original matter, and this of small importance, has been added, and it is given as an original production; of Dr. Bushe, it being merely said in the preface, that to him the author is indebted for much valuable information!

Let us now take a view of a portion toward the end of the book; the seventeenth chapter corresponds to the fifth of Bushe's work, the one being entitled Laceration of the Rectum; the other, Injuries of the Rectum.

Dr. Bushe says:—

"If the consequence of defecation, the rent is either transverse or vertical. When transverse, it is situated above the internal sphincter, and is the effect of the forcible extension of a fold of the mucous membrane, which, lapping under the mass of indurated fæces, becomes forcibly everted by their expulsion, and in undergoing this change of position is torn from side to side. When vertical, it generally terminates where the skin and mucous membrane unite, and is the result of forcible and violent distension of the anus. (NOTE.—In some cases of constipation, while the expulsive muscles act with great energy, the sphincter remains contracted and yields but slowly.") * *

Mr. Ashton says:—

"If the rent is the consequence of defecation, it may be either vertical or transverse; when vertical it results from undue distension of the anus during the violent efforts of the expulsive muscles, while the sphincters are contracted, or yield but slowly; it usually terminates at the line of junction of the skin and mucous membrane; when the laceration is transverse, the situation is above the margin of the internal sphincter, and is the effect of a fold of mucous membrane of the pouch of the rectum falling under a mass of indurated fæces at the time of their forcible extension, and being dragged down with them is torn from side to side."

By having changed the order in which Dr. Bushe treated the two forms of rent, Mr. Ashton has concealed, to some degree, the plagiarism, but by restoring this order it is quite apparent. We will make a few more extracts from the same chapters:—

"The pain, however, gradually but never entirely subsides, and is always renewed, with more or less severity, by each successive evacuation. * * Inflammation commences, effusion of

"Pain, which is lessened after a time, but does not entirely subside, and is renewed with more or less severity whenever the patient goes to stool. * * Inflammation is set up, lymph takes place, the edges and base of the rent become swollen, granulations sprout up, followed by suppuration, and now either cicatrization ensues or the wound is converted into an ulcer.

"The great object in the treatment of this injury is to keep the bowels easy by the use of emollient lavements and after each evacuation to cleanse the wound; for I have seen some cases in which the lodgment, even of a small quantity of feculent matter between the lips of the wound, created the most agonizing pain and spasm of the sphincter ani," etc. etc.

lymph is effused, the margins of the rent become swollen, granulation and cicatrization follow, or the wound degenerates into an ulcer.

"In the treatment of this injury it is essential to keep the bowels easy; emollient enemata will effect this object better than any other means. The wound must be cleansed after each evacuation, or the lodgment of particles of fecal matter will possibly give rise to agonizing pain and spasm of the sphincter," etc. etc.

We will give a few more extracts taken from the chapters on Laceration of the Rectum. These we give principally in order to show that when portions do not at first glance much resemble one another, a slight examination is sufficient to render this manifest.

Dr. Bushe says:—

"The symptoms of this disease are pain, a sense of weight in the sacral region extending to the loins, vesical irritation, tenesmus, the discharge of a thin bloody fetid pus from the anus, besmearing of the fæces with the same. (NOTE.—Mr. Colles says:—'At times the quantity of the discharge is much loosened, and then the sufferings of the patient are much aggravated; but on the flowing off of a larger quantity, he experiences great relief.' This I have not observed; indeed, in the last case specified in this chapter the pain has always been most severe, when the discharge was most abundant.) Smarting and even acute pain in the rectum invariably increased by defecation; and, when the ulceration extends low down, spasm of the sphincters with the other concomitants mentioned under the head of fissure.

Dr. Ashton says:—

"The symptoms of ulceration of the rectum are pain in the gut extending up the sacrum to the loins, or sense of weight in the bowel—a discharge of sanious, purulent or mucopurulent matter oozing from the anus,—the fæces will be besmeared with blood and pus, and the patient will be troubled with tenesmus, and irritation of the urinary organs. Mr. Colles, speaking of the pain and discharge in this disease, says:—'At times the quantity of the discharge is much loosened, and then the sufferings of the patient are aggravated, and on the flowing off of a large quantity, he experiences great relief.' This, I presume, must have been due to the acute and excessive inflammatory action, and not depending alone upon the quantity of matter secreted by the ulcer. Smarting at stool, and if the ulcer be situated near the verge of the anus, there will be spasm of the sphincter, as in fissure of the part.

"If the finger be introduced, the ulcerated surface may be detected by its roughness; but the better plan is to dilate the anus with the speculum, (NOTE.—Mr. Colles recommends a blunt, polished gorget, with its concavity looking toward the seat of the disease, to be passed upon the fingers into the rectum; then by everting the anus as much as we can, we shall obtain a full view of the ulcer by the light reflected from the gorget,) when the situation, extent, form and character of the disease can be easily determined. In many cases, however, nothing more will be necessary than to separate the buttocks, and press apart the edges of the anus with the fingers."

"If it exists higher up * * the speculum must be used to dilate the anus, when we shall with ease be able to judge of the situation, form, extent, and character of the ulcer. Mr. Colles recommends 'a blunt, polished gorget, with its concavity looking toward the seat of the disease, to be passed upon the finger into the rectum; then by everting the anus as much as we can, we shall obtain a full view of the ulcer by the light reflected from the gorget,' when * * it may be brought into view by divarication of the buttocks and pressing aside the edges of the anus with the fingers."

We might go in this way much farther, but it seems to us that we have shown sufficiently the truth of our assertion, that by merely stating in his preface, from Dr. Bushe's work he has gained much useful information, Mr. Ashton has not expressed with sufficient explicitness the extent of his indebtedness to it. By declaring that he had revised and extended a valuable work, which the author had been prevented by death from doing for himself, he would have spoken with more propriety. We prefer saying no more on the subject, but we feel that we ought not to leave it without having called attention to the manner in which the vast number of extraordinary cases that Dr. Bushe had collected with much laborious research, and printed in notes at the foot of the page, particularly on the subjects of abscess near the rectum, foreign bodies in the rectum, and hemorrhoidal affections, have been copied and inserted in the text of Mr. Ashton's book.

From the titles of the various chapters contained in the work before us, and which we have given above, it will be seen that all, or nearly all, the numerous affections to which the rectum and anus are liable, are treated of therein. There is but one, at least so far as we can recollect, that has been omitted, and that is the affection, first pointed out, we believe, by Dr. Physick, known by the name of *Preternatural Pouch* of the anus.* With this single exception the surgeon will find therein information, and

* This affection is treated of very fully by Dr. Reynell Coates, in the ably written article *Anus*, in the *American Cyclopedia of Medicine*.

valuable information, for every case of disease about the rectum and anus that will come under his notice. It might, perhaps, we may add, have been well to have left out the consideration of the subject of habitual constipation, and to have treated, in its stead, of the anatomy and functions of the rectum and of the anus.

It is scarcely possible to overrate the importance to the medical man of a proper knowledge of these affections, which Mr. Ashton, in his introduction, declares to be more prevalent, to cause more suffering, and to induce a greater number of varied and distressing symptoms than any other class of diseases, among civilized communities. In fact, as M. Begin says, the lower termination of the large intestine, and the opening which follows it, are endowed with every condition that could render their lesions at once frequent and serious. "A double ring surrounds the anus, which is opened only by the exertion of forces situated higher up. Large mucous follicles destined to favor the slipping of the matters to be expelled, a delicate sensibility often aroused, numerous arteries and veins forming a plexus, at times enormous, a reservoir wherein are accumulated substances irritating by their volume, their consistence, or their composition; in both sexes, the neighborhood of the most active portion of the genito-urinary organs, whose excitations, congestions, or pathological alterations extend readily to the contiguous organs; finally, the efforts of coughing, of speaking, of great muscular movements which react upon the anal region,—these are, (not all, for their enumeration would lead us too far,) but these are the principal circumstances of structure, of connection, and of function, that render the rectum and the anus so important in pathology." Of these affections, some are much more important and much more frequently met with than others, and consequently worthy of much more attention. Those to which most space has been devoted in this work are fissure of the anus, hemorrhoidal affections, prolapsus of the rectum, fistula in ano, stricture of the rectum, and malformations of the rectum, and we will enter into some consideration of the doctrines taught therein upon these several affections.

If the patient's suffering may be taken as the criterion of the importance of a disease, a comparatively lengthy chapter is very properly devoted to fissure of the anus and rectum, for in the whole catalogue of ills to which the human frame is liable, there are but few that give rise to as much suffering as this one, which is well styled by Mr. Syme, "that most intolerable of ailments." A distinction, however, exists in fissures of this region, and it ought to have been noticed by Mr. Ashton, who has omitted to mention it, as in fact have most writers on the subject; there are *painful* fissures, and fissures that are *not painful.* It is not every crevice about the rectum that will give rise to horrible pain; a surgeon, whose position at the Hospital du Midi, in Paris, had compelled him to make very frequent examinations of these parts, told us that he had many times observed fissures that were not at all painful and that did not reveal themselves by any symptom. Mr. Quain, therefore, in his very instructive work on diseases of the rectum, very properly styles this affection *painful ulcer*.

In the treatment of this troublesome affection, Mr. Ashton has much more confidence in the effect of local applications than is generally accorded to them by surgeons. He says: "My experience fully justifies me in stating that in the majority of recent cases it is not necessary to have recourse to an operation, although some of high authority in the profession assert that incision is the only effectual remedy, and that all sorts of applications, soothing or irritating, are unavailing," (p. 43.) We are inclined to believe that Mr. Ashton is correct in this opinion. One of the worst cases of fissure that ever came under our notice, in which division of the ulcer and of the sphincter ani had been performed without avail, and by one of the first surgeons of this country, was cured by the patient himself by the following course of treatment. He commenced by evacuating his bowels thoroughly with citrate of magnesia; using afterwards cold water injections; he then passed two weeks without having an evacuation, eating very little and of very light food; at the expiration of which time he repeated the citrate of magnesia and the enemata, and so on; at the end of two months he was well, and has remained so for more than a year. He took care, and this we believe to be very important, to have his stools in bed in a reclining position, and to remain there for twelve hours afterwards. We are acquainted with a second case in which this course of treatment proved efficacious, after an infinity of local applications had failed.

Mr. Ashton appears to us to pay too exclusive attention to the local treatment, only adding that, "at the same time it will be necessary to attend to the state of the secretions and excretions, and to correct any error in the patient's habits and manner of living," (p. 45.) The preparations he prefers as local applications are those of lead, opium, and belladonna, applied both as washes and as ointments. It is rather strange that he makes no mention of the use of infusion of rhatany in injection, for it has deservedly acquired great reputation.

The operation for the cure of fissure, described by Mr. Ashton, is a modification of the one recommended some fifty years ago by Boyer; it having been shown that an incision through the ulcer suffices for its cure, without dividing the sphincter. This incision may be made from within outward, or, as advocated by Mr. Syme, from without inward. Mr. Ashton expresses no preference for either of these methods; but having several times wounded himself when performing the latter operation, he finds the better plan to be to introduce the speculum and to cut into the open side. There is another operation, which we have seen performed a number of times, and which ought undoubtedly to have been noticed in this work; we mean forcible instantaneous dilatation of the sphincter, first performed by Récamier, and afterwards modified by Nélaton. The two thumbs are introduced, one after the other, into the rectum, the four other fingers of each hand being applied over the ischiatic tuberosities; in this position the thumbs are separated until they touch the internal face of the tuberosities. In doing this a resistance is felt, when suddenly an interior rupture is perceived, and the two thumbs touch the bone; this is sufficient. In this operation there is no danger of wounding the blood-vessels of the rectum, and consequently of purulent infection; nor is there any dressing to make. During the first few days it is well to administer injections, in order to facilitate the stools, and at the end of a week the patient can attend to all his occupations.

The chapter treating of *Hemorrhoidal Affections* is the longest in this book; it contains one hundred and fourteen pages, not one too many, however, when the frequency, and, oftentimes, the severity of these affections are considered. Frequent as these affections are, if the peculiarities of the venous circulation in the portal system be reflected upon, it must be quite a matter of surprise that affections of the veins about the rectum are not still more common. From the resistance the blood in the general circulation encounters in traversing the capillaries, much of its moving force is lost, and to move regularly in the veins it requires some assistance, that is obtained chiefly from muscular movements and the aspiratory action of the movements of inspiration; by neither of these is the flow in the portal veins affected. Besides, the portal veins possess no valves, and the blood contained therein is between two systems of capillaries, for these veins act as arteries in the liver, and the blood passes through a capillary net-work in that organ before it empties into the inferior vena cava. If, in passing through the general capillary system, the blood loses nearly the whole of its moving force, it is evident that the capillary system in the liver must act in the same way, and with so much the more power, as that the tension of the blood in the portal vein is already itself less than that in the arteries. In fact, well-known experiments, as for instance those made when the blood of the liver and that of the portal veins are collected for chemical examination, show that the circulation in the vena portarum is an entirely passive one; its current is directed toward the liver only on the one condition that there is a force to push it there, and this force comes from the abdominal walls, which, by pressing the mass of the intestines, propel the blood contained therein. Causes that may locally modify the circulation must, of course, act here with great efficacy, and it is far from being a matter of surprise that the hemorrhoidal veins, those most exposed, are often enlarged and diseased. Nature, as usual, has made provision for their protection, and were it not for the relief afforded them by the communications existing in the walls of the rectum, between the inferior mesenteric vein, and the middle and inferior hemorrhoidal, branches of the hypogastric, they would be still more often affected.

It is very hard to say how far the rapidity of the current of the blood in the portal vein may be diminished, but one experiment is sufficient to show the influence, in this respect, of causes constantly occurring. The ferro-cyanuret of potassium shows itself in the urine as soon as it reaches the general circulation; this salt, when given twenty-four minutes after a meal, appeared in the urine at the expiration of sixteen minutes; and when given two hundred and forty minutes after a meal, in two minutes. In fact, when the fatal consequences of the sudden injection into the general venous system of large quantities of even the most innocuous fluid is considered, the necessity of the prevention of the sudden passage into the inferior vena cava of the materials carried into the vena portarum, is at once apparent. Most important chemical changes undoubtedly occur in the liver, but it might be shown, we think, by reasons taken from numbers of facts in normal and pathological anatomy, both human and comparative, as also in physiology, that its action as a mechanical agent is most important. If a few ounces of water suddenly thrown into the general circulation of a dog can produce death, what would be the effect if, in man, the liver did not intervene between the inferior vena cava and a barrel of lager beer?

Hemorrhoids have been divided into many classes by some writers, but their usual classification, as internal and as external, the one adopted by this treatise, is all that is necessary for practical purposes. As Mr. Ashton says:—

"The most practical and important classification to bear in mind, as influencing the treatment, is the division adopted by most English authors into internal and external hemorrhoids; the former being those which occur within the margin of the anus, and involve the mucous membrane of the intestine, and the latter, those which are situated external to the sphincter ani, and are covered by the thin integument of the anus," (p. 87.)

As to the pathological anatomy of these tumors, external hemorrhoids are said by Mr. Ashton—

"To consist of the integument and cellular tissue, into which the blood has been extravasated, as a result of a congested state of the hemorrhoidal vessels, and determination of blood to them, produced by causes to be hereafter mentioned; generally the blood is encysted in a central cavity, with a smooth, glistening surface; in some cases there are several of these cavities filled with blood," (p. 88.)

The tumors constituting internal piles, he says—

"Consist of a morbid alteration in some portion of the mucous membrane of the rectum, and submucous areolar tissue, with an augmented and abnormal development of the capillary vessels," (p. 90.)

"By dissection, internal hemorrhoidal tumors will be found to consist of both arteries and veins, the latter capacious, not in a diseased condition, but merely of abnormal development; the areolar tissue of the mucous membrane is hypertrophied, and if the tumors have existed long, and been subject to repeated inflammatory attacks, it will also be condensed. To the touch they have a spongy elastic feel, and by some authors are considered to resemble erectile tissue in structure; had they compared them to those abnormal developments of the vascular system termed aneurism by anastomosis, the analogy would have been more correct," (p. 91.)

We must differ from Mr. Ashton as to the nature of internal hemorrhoids, although so confident is he of the correctness of his own opinion, that he declares it somewhat surprising that many of the later writers should hold a different opinion in regard to them. Internal hemorrhoids do not always leave any trace after death, and when an injection is thrown into the mesenteric or the portal vein, they make their appearance again; they, therefore, must be varices. The dissections of Blandin and of Jobert show, to our mind most clearly, that primitively these hemorrhoids are varices. When they have lasted long, the causes from which they had their origin continuing to act, changes occur in them, masking their origin; from adhesive phlebitis the walls of the veins thicken; or becoming thinner from pressure, they yield, and blood is effused in the neighboring cellular tissue, which becomes hard and thickened from inflammation thus excited. At the dissection of an old hemorrhoid, the veins are found having lateral dilatations, full of blood, and sometimes of clots, which may be incrusted by calcareous matter, and form true phlebolithes; the cellular tissue between the veins is indurated by the presence of granular, amorphous matter, which renders the fasciculi very adherent, and containing some fibro-plastic elements and elastic fibres. The old opinion of Morgagni, who says, "Hæmorrhoides nihil esse aliud quam varices venarum ani," is always true of them, when internal as well as external, at least when recent.

Under the list of causes giving rise to hemorrhoids, there is nothing mentioned in this work which has not often been given before.

"Any impediment," it is said, "offered to the return of the blood from the lower bowel will cause hemorrhoids. It will arise from two causes, the one being mechanical in its immediate effect, the other pathological, and depending on disease and alteration of structure in some of the internal organs. Those causes which act mechanically are the pregnant uterus, ovarian and other tumors developed in the pelvis or abdomen, which, by pressure on the large venous trunks impede the ascent of the blood; tight-lacing and cinctures also have the same effect. The pathological causes are congestion and structural diseases of the liver, pancreas, and spleen; diseases of the lungs, heart, and large blood-vessels, interfering with the free circulation of the blood," (p. 110.)

A cause of hemorrhoids, at least what in our way of thinking may be one, which has been neglected by Mr. Ashton, and by all other writers on this subject with which we are acquainted, is weakness or relaxation of the muscles of the anterior wall of the abdomen. It is, as was said before, undoubtedly a fact, proved by experiment, that the pressure exerted by these muscles upon the mass of intestines is the cause of the onward movement of the blood in the portal veins toward the liver, and any cause diminishing this pressure, whether position of the body or feebleness of the muscles themselves, must lead to more or less stagnation of this blood, and consequently to hemorrhoidal affections. Tailors, shoemakers, dressmakers, and persons engaged in writing, which forces them to lean forward over a desk, are particularly subject to hemorrhoids; and we would explain the well-known influence of sedentary occupations generally upon these affections more by their producing relaxation of the anterior abdominal walls than by the deficiency of exercise and the constipation attending them, though these must play their part also. An undoubted cause of an attack of hemorrhoids not mentioned by Mr. Ashton, and one not unfrequently witnessed, is parturition. It is not very uncommon for the patient to suffer most severely after the birth of the child, from the strangulation of a mass of hemorrhoidal veins that have been forced out with the first discharge of fæces, and unless they are recognized and speedily replaced, they give rise to intense suffering at a time when the system is not most able to endure it. As the abdominal walls are in a state of extreme relaxation, the fact that hemorrhoids are often seen at this time, is one important to notice, in connection with what has just been said.

The diagnosis of hemorrhoids is attended with but little difficulty, for there are few diseases with which they can be confounded. In case it may be desirable to arrive at a correct knowledge of the tumor, and of the condition of the neighboring parts, a digital examination of the interior of the bowel must be made. In making this, previous to the introduction of the finger, Mr. Ashton advises that—

"The cavity of the nail should be filled with soap, which will prevent it scratching the intestine, and the finger must be dipped in oil to facilitate its introduction; lard and unguent do not answer so well, as they interfere slightly with the delicacy of the sense of touch." As to the position of the patient, "he should be directed either to lean over the back of a chair, or to lie upon a sofa on his side, with the nates projecting over the edge, and the knees drawn up; the latter position is preferable, and should always be adopted with female patients," (p. 122.)

Before proceeding to any local treatment for the cure of hemorrhoidal tumors, it is always proper for the surgeon to examine as to the safety of arresting the discharge by which they are accompanied. Many surgeons have no hesitation in so doing; Mr. Syme, for example, "protests against the mischievous fallacy of representing such a drain as salutary;"* but cases in which its arrest has been followed by severe and even by fatal consequences, are recorded in treatises of unquestionable authority. Mr. Copeland, in his "Observations on the Principal Diseases of the Rectum and Anus," (third edition, London, 1824, p. 57,) cites several cases that had come under his notice, where the consequences were most alarming, and one in which death was caused by the suppression of the discharge.

Mr. Ashton's views upon the subject are very correct, and we will give them in his own words:—

"When piles have existed for a long period, have continued from youth, or the commencement of puberty, when they supervene upon or replace some serious organic or habitual affection, if they are preceded by constitutional disturbance, and succeeded by an improvement in the state of the health, if well-marked indications of plethora exist, which are relieved by the accession of the hemorrhoidal flux, and if indications of congestion, or disease in any of the organs, accompany or follow its suppression or interruption, or any hereditary predisposition exists, a constitutional nature may be inferred, and local treatment must be a secondary consideration, and not adopted till the constitutional cause has been removed or palliated; this is especially necessary if there is a predisposition, hereditary or otherwise, to apoplexy, gout, phthisis, hæmoptisis, epistaxis, or other kind of hemorrhage," (pp. 122 and 123.)

Both the constitutional and local measures to be used for the relief of a patient suffering under hemorrhoids and the various complications to which they are liable, advised by Mr. Ashton, are very judicious, and they are detailed with great minuteness. In case the removal of the tumors be decided upon, he prefers to excise them, if external, and when internal, to make use of the ligature, and in some cases, of the nitric acid. The fatal results following the application of the ligature, such as phlebitis, diffused inflammation of the cellular tissue of the pelvis, peritonitis, and tetanus, he refuses to credit. Although instances have been recorded by surgical writers, he declares that—

* On Diseases of the Rectum, by James Syme. Third edition. Edinburgh, 1854, p. 14.

"They have neither verified their surmises as to the cause of death, by post-mortem examination, nor have they shown that the cases were such as justified surgical interference," (p. 137.)

The plan we have usually seen adopted for the removal of hemorrhoidal tumors, is their destruction by the actual cautery, a mode of treatment which we do not find noticed by Mr. Ashton. The patient being placed upon a table, a metallic wire is first passed through the tumor so as to hold it properly, and the mass is then thoroughly burned, with the red-hot iron, the neighboring parts being protected by a thick fold of linen, dipped in water, having a hole in the centre corresponding to the structures to be cauterized. By taking this plan the surgeon runs no risk of phlebitis, which is certainly to be feared after applying ligatures to veins; they should never be used when they can be avoided. Whatever may be the local treatment adopted, after the removal of the hemorrhoids, the patient must carefully avoid all the causes that induce the disease under which he had labored.

As usual, to this chapter upon hemorrhoidal affections is appended a large number of cases, for the purpose of illustrating the different phases and the treatment of the disease therein described. In this instance as many as forty-two pages are thus occupied.

The subject of *Prolapsus of the Rectum*, which occupies the eleventh chapter, is discussed in rather an unsatisfactory manner, particularly as regards the treatment of that affection. Mr. Ashton advises the adoption of some measures which may, we think, be even injurious, and omits all mention of others by which we have known the condition of the patient to have been materially benefited. We doubt, for example, the utility of emollient enemata which are recommended by him for the purpose of preventing costiveness, (p. 210,) and are strongly inclined to partake the opinions of Mr. Quain, in respect to the use of injections of any description in the treatment of prolapsus. In the excellent work which we have already cited, after relating a case of long-continued prolapsus cured by operation after the failure of other means, among which were the injections of the tincture of the sesquichloride of iron, so long recommended by surgical writers, Mr. Quain adds:—"As regards the fluid injected in this instance, I cannot say that I am disposed, from what I have seen, to expect advantage from it, or, indeed, from any other used in the same way. As, moreover, the injections bring on fresh action of the bowels, I think it on the whole best to abstain from using them," (p. 135.) When an operation is had recourse to for the relief of the patient,—if he be more than five or six years of age, so far as we know, all other means fail to cure,—the one preferred by Mr. Ashton, as the most simple, most effectual, and the least painful, is that performed by Mr. Copeland.* In this operation a number of ligatures are applied, for the purpose of strangulating the prolapsed portion of bowel. In this edition of the work he also recommends, for the first time, the application of the concentrated nitric acid, which he says he has made use of "with the happiest result, and thinks it the better plan of treatment, except in those cases in which the mucous membrane is very lax and voluminous," (p. 213.)

An operation we believe to be preferable to either of these, but not mentioned by Mr. Ashton, is the destruction of the prolapsed mucous membrane by means of the actual cautery, in the way described when speaking of hemorrhoids. We must express some surprise that this operation is not mentioned in either of these chapters, for it might be said to be the only one now performed for the cure of piles and of prolapsus ani, by continental surgeons. The only objection to its use is the pain it causes, and Mr. Ashton says that when his patient prefers to take chloroform, he does not refuse to administer it, (p. 156.)

Another operation recommended by Mr. Ashton, when the protrusion is the result of relaxation of the anus, is the excision of a marginal fold of the integument and mucous membrane from either side. This plan was first recommended by Dupuytren; at least that surgeon practiced it very frequently, and considered it a very successful one. M. Nélaton, who was his interne at the Hotel-Dieu, made it a habit to follow the patients who left *cured;* and he found that after they had recommenced to work, th affection almost always returned.

We have known adults suffering under prolapsus of the rectum, to be rendered quite comfortable by pursuing a very simple plan, which we do not find mentioned by Mr. Ashton. It consists merely in having the daily evacuation of the bowel, just before retiring to bed, instead of, as is usual, early in the morning. Patients sometimes find this out for themselves, that if they go to stool in the evening, and immediately afterwards go to bed, the rectum does come out, and they can attend to their affairs very comfortably the following day.

Mr. Ashton pays considerable attention to the constitutional treatment in these cases, and it is of the utmost importance, particularly when the patient is young. In such patients as a general rule, the whole of the intestinal mucous membrane is inflamed, as well as that portion of it protruded at the anus. This portion, in fact, is only protruded mechanically from the continued straining, which is caused by the frequent stools induced by the diseased condition of the whole intestinal canal.

*Fistula in ano*, the subject of the thirteenth chapter, is, after hemor-

---

* *Loc. cit.* pp. 79 to 83.

rhoidal affections, the one to which in this work Mr. Ashton has devoted the greatest number of pages, fifty being thus occupied.

As respects the formation of the internal opening in complete fistula, there are, as is well known, two opinions, each held by able men equally positive as to their own correctness. Mr. Syme, for instance, expresses himself as follows, the italics being his own: "Having made this examination times innumerable, before as well as after evacuation of the abscess, I do not hesitate to affirm, that when a *fistula in ano is formed, the mucous membrane always remains entire in the first instance, and is never perforated until after suppuration has taken place*," (*Loc. cit.*, page 25.) Sir Benjamin Brodie, on the other hand, says: "I believe that this is the way in which fistulæ in ano are always formed, namely, the disease is originally an ulcer of the mucous membrane of the bowel, extending through muscular tissue into the cellular membrane external to the intestine," *Clinical Lectures on Surgery, etc.;* Philadelphia, 1846, p. 187.) From cases that have come under his own observation, Mr. Ashton differs from the authors just quoted, as to the internal opening being always formed either in one way or the other; he is convinced that the perforation of the intestine takes place in both the ways just mentioned, (p. 238.) This is, at all events, the opinion that most simplifies the affair.

This internal orifice, as was first pointed out by M. Ribes, is always situated very near the margin of the anus; in seventy-five cases examined by him, after death, in not a single instance did he find it situated at a greater distance from the anal margin than five or six lines. Of course, in exploring with the probe for the purpose of discovering the situation of this orifice, it is very necessary to proceed with the greatest gentleness, bearing in mind at the same time its usual position. On this point Mr. Ashton judiciously remarks:—

> "In making the exploration, no force should be applied to the probe, or it may be thrust through the walls of the sinus into the loose cellular tissue surrounding the gut, and a very erroneous impression of the course of the fistula obtained. It must be recollected that a probe is an instrument not to be directed with an absolute control, but one from which we are to gather information: it is to guide and instruct us," (p. 243.)

It is at times exceedingly difficult to find this internal orifice, and in such cases we have seen the expedient of injecting an innocuous colored liquid, as milk, into the external orifice, made use of with success; the liquid finds its way to the internal orifice whence it can be seen to flow. We have seen this same expedient successfully resorted to also in a case of vesico-vaginal fistula, where the orifice was so small as to be detected with difficulty; by throwing milk into the bladder through a catheter, it was seen dropping into the vagina, into which, of course, a speculum had been introduced.

"If," says Mr. Ashton, "the health of the individual is good, and all circumstances are favorable, a fistula may sometimes be made to heal without an operation," (p. 249.) This is all very true, but it is not sufficiently explicit; what these circumstances are that are favorable ought to have been mentioned, otherwise the surgeon will lose time and probably his patient, in attempting an impossibility. When the fistula is only separated from the anus by the thickness of the mucous membrane, it will be vain to attempt to heal it without an operation; but when a considerable thickness of tissue intervenes, this can often be done. In the one case, the wall of the fistula is movable, but in the other it is solid, fixed, so to speak, in the perineum. The injections to be used for this purpose, mentioned by Mr. Ashton, are solutions of the sulphate of zinc or copper, or the nitrate of silver; that which we have seen used with success, and which we should prefer to either of these, is composed of one-third of the tincture of iodine and two-thirds of water, with some iodide of potassium to hold the iodine in solution. When an operation must be performed for the cure of the fistula, Mr. Ashton well describes the mode in which it should be done, as follows:—

"On the morning previously to the operation, the bowels should be acted upon by a mild cathartic, and an enema of warm water or thin gruel administered; the operation is then to be performed in the following manner:—The patient kneeling on a chair, and resting on the back of it, or leaning with his elbows on a table, or lying on a bed or couch, with his knees drawn up, and the nates projecting, an assistant separates the buttocks, and the surgeon, introducing the forefinger of the right or left hand into the rectum, according to the side on which the fistula exists, makes himself familiar with its track and position by using the probe as previously directed; having accomplished this, he passes the blade of a probe-pointed curved knife into the external orifice along the course of the fistula, making it emerge through the internal opening, the point being hitched by the finger in the rectum; both hands are then depressed, and with a slight sawing motion the intervening tissues are divided, and the knife and finger brought out together. If the surgeon be timid, or unaccustomed to operate, or the fistula so tortuous that the knife cannot readily pass along its track, a grooved silver director, or strong probe, may be used; it must be bent as required, and, having been introduced through the opening in the integument and that in the bowel, the end is pulled down, and made to protrude at the anus; the parts are then to be divided by passing a sharp-pointed curved bistoury along the grooved channel, and the operation is finished," (p. 260.)

The measures to be adopted after the operation, and until the complete healing of the wound are also carefully detailed. When the fistula is of the blind internal form, it is advised to make it complete by passing a bent probe into the sinus, and then cutting down upon the end projecting against the integuments; the operation can then be finished with a curved knife as just described. In the same way, an external blind fistula, extending to the coats of the intestine must be made a complete one by perforating the bowel with the knife used in operating for complete fistula, by pressing it against the edge of the nail of the forefinger, previously introduced into the bowel; the operation is then at once finished in the usual way. By thus presenting the edge of the nail to the edge of the knife, instead of the pulp of the finger, the surgeon will not wound himself, and the plan is a more convenient one than that of using a wooden gorget in order to avoid this accident.

It is sometimes necessary in operating for the cure of this affection, to divide parts so deeply seated that their destruction by a cutting instrument would not be without danger. Cases of this kind are not noticed by Mr. Ashton. When they are met with, the parts may safely be divided by the forceps devised by Dupuytren, in his operation for the cure of artificial anus; so great a constriction of the parts inclosed being produced by them, that circulation becomes impossible, and they mortify. This plan, though successful, is attended with the inconvenience of forcing the patient to wear an instrument for some time, perhaps four, five, or six days, projecting from the anus. Instead of seizing the part to be destroyed by an instrument, thus slowly producing its destruction, M. Nélaton seizes it in a pair of hot forceps, and destroys it in a few moments. In performing this operation the neighboring parts are protected by the gorget made use of in the operation of lithotomy.

When the patient laboring under fistula is the subject of phthisis, Mr. Ashton agrees with most surgeons in dissuading from surgical interference. He says:—"If the operation be performed, the wound will not heal; or should it do so, the probable result will be either the formation of a fresh abscess, or the aggravation of the pulmonary disease," (p. 266.) He adds, however, very properly, that he should not refuse to operate, if by declining he should induce a sense of hopeless mental depression and despondency.

In the chapter upon *Stricture of the Rectum*, Mr. Ashton treats of contractions of the gut that are the result of other than malignant diseases. It is a disease not often met with, but one causing great annoyance; and although not attended with immediate danger to the patient, yet he becomes gradually exhausted. It is, as Dr. Bron says in an excellent little treatise on the subject,* "It is with strictures of the rectum

---

* Nouvelles Considérations sur les Rétrécissements du Rectum, par le Dr. F. Bron; Lyons, 1856.

as with strictures of the larynx; the patient who has a stricture of the respiratory passages does not always die of asphyxia, but he gradually dies out, because he only breathes a part of what he should. Hæmatosis is insufficient, nutrition and innervation are enfeebled, and the patient dies slowly. Tormented by the idea of passing the food they take, patients often take too little; they stint themselves, and the insufficient alimentation, joined to the delay of which we have spoken in going to stool, at last always leads to disorders in the digestion, in the circulation, and in the innervation, and renders more serious the prognosis which the practitioner must give."

After enumerating a great number of possible causes of stricture, Mr. Ashton concludes by saying that "authors also mention syphilis, metastasis of cutaneous eruptions, and suppressions of discharges that have existed for some time, and have become habitual, as causes of stricture of the rectum." This is not allotting sufficient importance to syphilis as the cause of this complaint, which it is important to do, particularly on account of the influence of anti-syphilitic remedies in its cure.*

The most usual seat of the disease he agrees with most authorities in placing at a distance of not more than two or three inches from the anus, where it can readily be detected by the finger. Just at this spot in the rectum a quite well-marked muscular circle can always be found existing external to the longitudinal muscular fibres of the intestine; and the stricture has been shown to be, in some cases, owing to the hypertrophy of this muscular bundle—an important fact not mentioned in this treatise.

In the treatment of this affection the object to be obtained is, of course, to restore the bowel to its original dimensions, or, at least, to enlarge it sufficiently to permit the fæces to pass with freedom. Dilatation by means of bougies is the only plan for effecting this purpose that Mr. Ashton recommends. When the disease is the result of chronic inflammatory engorgements, by compressing the swollen parts, simple dilatation would be effectual. When, however, it is the result of contraction, from hypertrophy of the muscular tissues, such treatment will not be sufficient; and sudden and violent dilatation, by the tear it produces, will be required in order to bring about a cure. Some surgeons have found that by effecting dilatation with the bladder of a calf, the patient suffers less pain and inconvenience than when the ordinary bougie is used, as if the contact of animal matters with our tissues is more supportable than any other substance whatever. Of course when the result of syphilitic infection of the system, proper constitutional treatment will be demanded; but not only in

* See the Memoir of M. Gosselin on this point in the *Archives Gén.* for 1852, 5ième séries, tome iv.

such cases is constitutional treatment beneficial, in many others it is very important, and we must consider it strange that Mr. Ashton, who, as a general rule, is particularly attentive to the constitutional treatment, should here say nothing about it. Though local measures in the treatment of stricture are of great importance, they must, by no means, be regarded as the only, or as the chief resource. If, as Mr. Quain remarks, the eye, or a serous membrane, or the testis be affected with fibrinous deposits, medicinal treatment is, by universal assent, resorted to; and if the judicious use of certain medicines be useful in such cases—and it undeniably often is so—why should the fibrinous deposit in the bowel be considered to be necessarily beyond the reach of similar means?*

We have now finished the task we had allotted to ourselves, and have examined at considerable length those chapters in Mr. Ashton's book to which he has devoted most space and most attention. We undertook this task, be it understood, because his work is considered, and correctly so, the most complete one in the English language on the subject of which it treats. The book is quite a good one, but it could certainly have been made far more so, had Mr. Ashton, in addition to the writings of authors using the English language, been acquainted with the practice and publications of the French surgeons.

